# Postmitotic Regulation of Neuronal Subpopulations in the Cerebellum

**DOI:** 10.64898/2026.08.09.743777

**Published:** 2026-08-14

**Authors:** Pamela Valnegri, Shin-ichiro Fujita, Tomoko Yamada, Yue Yang

## Abstract

Hundreds of neuronal cell types and subtypes have been identified through transcriptome profiling of the mammalian brain and are thought to arise from lineage-restricted neural progenitors during early development. However, how neurons might further diversify their transcriptional identities during postmitotic development and through adulthood remains poorly understood. Here, we combine genetic and biochemical approaches to uncover granule neuron subpopulations in the anterior cerebellum, which we term C1 and C2. We find that a large proportion of granule neurons predominantly express the C2 gene program during early postmitotic differentiation, but that C2 genes are downregulated in a subset of neurons during late postnatal development, leading to comparable proportions of C1 and C2 neurons in adulthood. Using an *in vivo* genetic mini-screen, we identify calcium signaling pathways, together with the transcription factor ETV1, that establish the transcriptional program of C2 neurons. Finally, C1 and C2 granule neurons are differentially engaged during behavior and this reflects their roles in cerebellar-dependent associative learning. Together, these findings reveal postmitotic mechanisms that continue to shape neuronal identity in the brain.

## Full Text Availability

The license terms selected by the author(s) for this preprint version do not permit archiving in PMC. The full text is available from the preprint server.

